# The Handicap of the First-Born

**Published:** 1915-04-10

**Authors:** 


### THE HANDICAP OF THE FIRST-BORN.

### The Small Family and Racial Progress.

Whether or not one agrees with the doctrines, eugenical and other, which Professor Karl Pearson propounds, it is impossible to deny the ingenuity of his advocacy, the immense pains he takes to prepare his case, and the fascination both of his topics and of his presentation of them. In all his researches it is as clear as daylight that he seeks truth alone, and employs his very best energies and abilities in the quest. No labour is too great for him if it will serve to exclude a possibility or in any other way to close the last loophole of escape from any conclusion to which the facts as he sees them may be driving him. This unswerving scientific rectitude and the originality of his genius account, no doubt, for the devotion of his band of disciples and co-operators, and for the ever-growing influence of the Galtonian eugenic school, of which he is both the nominal and the real head.

### Health and the Order of Birth.

In a recent monograph * Professor Pearson reverts to a very interesting topic which he partially investigated some years ago—namely, the question whether there is any difference physically, mentally, or constitutionally between the first, the second, the third, and the several succeeding members of each family of the human species. It has been a generalisation accepted for a long time that on the average the second-borns are a trifle better equipped by Nature than the third-borns; that these in turn surpass slightly the fourth-borns; and that the first-borns are about at that lower level where the seventh-borns are situated. The hypothetical explanation of this alleged order of precedence was not difficult. Admittedly the birth of a first-born child is more prolonged, more likely to be attended with difficulty, and more likely to result in still-birth than are the births of subsequent children. Thus the chances of birth-injury, not fatal but sufficient to handicap the child physically or mentally, are definitely greater for the first-born. Add to this that the feeding and upbringing generally of a first-born is more in the nature of an experiment than that of subsequent children, owing mainly to the inexperience of the parents, especially the mother, and it is comprehensible enough that there may be environmental influences acting detrimentally, comparatively speaking, on the first-born which affect the younger children decidedly to a lesser extent.

### The Small Family.

The important question is, however, whether the first-born is actually handicapped in the struggle for existence; and, if so, to what extent, and in what particulars? To this question Professor Pearson has applied himself, and his results are full of interest. The problem he set himself to solve is complicated by a very great many intercurrent factors, which have to be untangled from the influence of the pure and simple order of nativity. Thus the handicapping of the first-born in small families may be partly due to the inclusion of many small families in which the first-born is also late-born; that is, the first-borns of women who marry at forty (say) may be especially handicapped because their mothers are nearing the end of the reproductive period; and such women are all the more likely to have but one child. Obviously the influence of a factor such as this must be discounted in considering the chances of the first-born to parents who marry at younger ages. So, too, there is another factor which weights the small families; they may be due to exhausted virility in the parents. Certain types of parental degeneracy seem incapable of producing more than one or two children, and the children of such parents are themselves feeble. Families such as this contribute much more to the mass of first-borns than to that of second-borns, third-borns, and later-borns; but it would obviously be unjust to ascribe the (average) handicap of the first-borns to purely ordinal considerations when part of it is due to parental degeneracy such as this. And there are many other disturbing elements in the problem which require to be sifted out before results can be reached which are unassailable. To follow Professor Pearson in detail through the mathematical mazes necessary to this purpose is unnecessary here. Suffice it that his methods, though they may not satisfy all his critics, are extremely thorough and conscientious.

### Statistical Evidence.

To start with, he shows by statistical tables that the proportion of still-births is twice as high amongst first babies as amongst second; and that it is lower still amongst third children. The percentage rises again for the fourth and succeeding children: for the seventh and over children (all lumped together) it is fractionally higher than for the second children, but still only a shade over half the rate for first children. Infantile death-rates during the first year of life tell much the same tale both among the professional classes and among the proletariate.

Proceeding next to idiocy, he is able to affirm from an analysis of statistics from the Royal Albert Asylum and from the Syracuse State Asylum that Mongolian idiocy affects in vastly greater proportion the last-born than the earlier members of a family: the first-borns are less liable to this form of idiocy than any of the other children, a result attributed to a possible exhaustion of fertility as a cause of Mongolian idiocy. Excluding Mongols from the statistics, the first-borns supply a considerably larger percentage of idiots than the second-borns, and the intermediates generally are even less likely to be idiots than the second-born; there is also some excess among the two last-born, though not so marked as among the first-born

* *On the Handicapping of the First-born.* By Karl Pearson, F.R.S. (London: Dulau and Co. Price 2s. net.)

With regard to epilepsy, the handicap of the first-born is not so marked, though it does exist. Professor Pearson is disposed to admit that while there is a weighting of the elder-born even in epilepsy, this is due to selection of families rather than to a selection of the elder-born in each individual family. As for insanity, the conclusion is very similar—namely, that there is a distinct bias against the elder-born, though it is not very great.

Albinism is another of the handicaps against which the first-born has to contend. The critical analysis of 952 albinos of European races shows a very definite excessive proportion amongst the first-borns. Criminality is yet another case in point. Here the handicap affects both the first and second born about equally, whereas the later-born children supply less than the due proportion of criminals relative to their numbers among the population. Tuberculosis is yet another of the bugbears of the early-born. According to Professor Pearson's statistics and interpretations of them, there is a definitely greater liability to tuberculosis amongst the first-born, and this exceedingly unpleasant disability is shared to some extent by the second-born; the third and later born children are all less likely to develop (clinical manifestations of) tuberculosis than are the first two of each family. Congenital cataract, a disease of a very markedly hereditary character, is also shown to affect the first-born unduly often.

The Apparent Moral.

The applications of these facts, if facts they indeed be, are of enormous importance. The handicap with which Nature loads the first-born means that the small family, so much on the increase among civilised nations, is detrimental to race progress. Professor Pearson is strongly convinced that the present tendency (exhibited more noticeably in France than anywhere else) to make the first-born 50 per cent. instead of about 22 per cent. of the whole number of births, spells degeneracy. "The individual feelings of the first-born, even if the handicapping were far more substantial than it is, cannot be considered to outweigh the national importance of the problem. If this principle of the handicapping of the first-born be true, as I have little doubt that it is—and if a similar principle holds for the last-born (to a lesser degree, it is true) for some conditions like Mongolian idiocy—what must be the moral? . . . Surely, that the better born are the intermediates in families from five to eight; and that when families are restricted to twos or threes, or extended to twelves and thirteens, there may be a quite appreciable tendency to increase the proportion of the less efficient in the community."

